# Analgesic treatment of refractory cancer pain caused by bone and soft tissue metastasis of lower esophageal and cardial adenocarcinoma: A case report

**DOI:** 10.1097/MD.0000000000042433

**Published:** 2025-05-16

**Authors:** Jiying Tang, Feng Ding, Mincheng Yu, Xiaojun Cai

**Affiliations:** a Department of Oncology, Renmin Hospital, Hubei University of Medicine, Shiyan, China; b Law Sau Fai Institute for Advancing Translational Medicine in Bone and Joint Diseases, School of Chinese Medicine, Hong Kong Baptist University, Hong Kong, China; c Institute of Medicine and Nursing, Hubei University of Medicine, Shiyan, China.

**Keywords:** adverse reactions, case report, opioid medications, pain management, refractory cancer pain

## Abstract

**Rationale:**

Pain management in patients with malignant tumors and concomitant bone and soft tissue metastases remains a significant clinical challenge, with 10% to 20% experiencing refractory cancer pain. These patients often present with multiple comorbidities and abnormal biochemical markers, necessitating a multimodal approach to therapy. The personalized application of patient-controlled analgesia (PCA) technology has shown promise in enabling swift and effective pain management.

**Patient concerns:**

A 66 year old male patient with metastatic lower esophageal cardia cancer was hospitalized due to uncontrollable right chest and back pain. Effective control was achieved through multimodal comprehensive treatment, especially the application of PCA technology. At the same time, the patient also received the opportunity of antitumor therapy.

**Diagnoses:**

He was diagnosed with (1) stage IV cardial adenocarcinoma of lower esophagus (rT3N0M1); (2) secondary malignant tumor of bone; (3) refractory cancer pain (numerical rating scale score 6 scores); (4) chronic viral hepatitis B; (5) hepatic insufficiency; (6) incomplete intestinal obstruction; and (7) chronic erosive gastritis.

**Interventions:**

The complete process of cancer pain treatment for a patient with lower esophageal and cardia cancer was analyzed. The titration and rotation of opioid dosage, adjuvant treatment of underlying diseases, prevention and treatment of drug-related adverse reactions were introduced, with emphasis on PCA rapid titration.

**Outcomes:**

The patient’s cancer pain is well controlled, with mild adverse reactions and timely treatment. In addition, the patient also received antitumor treatment (radiotherapy and targeted therapy).

**Lessons:**

This case underscores the importance of comprehensive evaluation and precise diagnosis in cancer pain management, highlighting the need to address underlying conditions, conduct multidisciplinary consultations, and develop personalized analgesia plans. Such approaches can enhance pain treatment efficacy, minimize adverse reactions, and improve the overall quality of life for cancer patients.

## 1. Introduction

Cancer pain is a common clinical symptom, and the incidence of cancer pain in patients with advanced tumors reaches 70% to 90%.^[1]^ In recent years, with the continuous progress and development of palliative care in China, World Health Organization (WHO) three-step analgesic treatment principles and National Comprehensive Cancer Network (NCCN) guidelines for adult cancer pain have been widely used.^[2]^ Eighty to ninety percent^[3,4]^ of the pain symptoms of cancer patients can be relieved by standard and effective treatment. However, there are still 10% to 20%^[5,6]^ patients with pain that is difficult to control or adverse reactions are difficult to tolerate, which belongs to refractory cancer pain. In 2017, the Refractory Cancer Pain Science Group of the Cancer Rehabilitation and Palliative Care Professional Committee of the Chinese Anti-Cancer Association published the expert consensus on refractory cancer pain,^[7]^ which clarified the definition of refractory cancer pain: It refers to the moderate and severe pain caused by the tumor itself or the factors related to tumor treatment, and the pain relief of the patients is still unsatisfactory and (or) the adverse reactions are intolerable after 1 to 2 weeks of standardized drug treatment. This consensus provides a new basis for the treatment of refractory cancer pain in Chinese population. Refractory cancer pain has a long duration and high intensity, causing great pain to patients, affecting their daily life and work, often combined with psychological problems such as anxiety and depression, further aggravating the pain, and clinical treatment is difficult. Treatment methods for refractory cancer pain^[7–11]^ mainly include nerve block, epidural administration, patient-controlled analgesic (PCA) analgesic pump, nerve destruction, etc. Among them, PCA technology has advantages in rapid titration of opioids and sustained treatment of refractory cancer pain that other treatment methods do not have.^[7,10]^ For example, PCA can maintain stable blood drug in order to reach continuous relief of moderate and severe pain, and can be used for dose-titration to overcome patient-specific differences, also PCA’s self-management can timely control breakthrough pain, the features of simple and convenient can effectively reduce the workload of medical care too. However, due to objective historical conditions reasons, PCA treatment of cancer pain is not known by local doctors. In 2021, the county-level medical institution where the patient is located does not have an independent oncology department or pain clinic, and other local specialists do not understand it, so they do not recommend this technology, resulting in a similar large number of patients with refractory cancer pain in remote areas without the opportunity to obtain PCA application. At the same time, due to the reasons of patients or doctors, there are a certain number of patients with refractory cancer pain in non-tumor and non-pain departments of municipal and prefecture-level medical institutions, but their doctors do not know much about PCA. All of the above factors have limited the promotion of PCA technology to benefit a small number of patients with refractory cancer pain. PCA technology, as one of the important treatment methods for refractory cancer pain, is recommended by the guidelines of NCCN^[2]^ and China’s refractory cancer pain expert consensus.^[7]^ It has many advantages as mentioned above and should be widely used clinically, but it faces challenges in the promotion of some prefecture-level medical institutions and most county-level medical institutions in underdeveloped areas. This study carried out the whole process management of refractory cancer pain in a patient with lower esophageal cardiac cancer complicated with bone and soft tissue metastasis. The comprehensive treatment process of cancer pain was presented. In particular, the application process of PCA technology was introduced in detail, and achieved good results with low adverse reactions. The personalized application of PCA technology has enabled patients to manage their cancer pain swiftly and effectively. Promoting the use of PCA technology among patients with refractory cancer pain holds great practical significance.

## 2. Case presentation

### 2.1. General information

A 66 year old male underwent partial esophagectomy, intrathoracic esophagogastrostomy, and regional lymph node dissection in October 2018 due to obstruction 3 months before eating. Postoperative pathological tips (lower esophagus cardia) adenocarcinoma, moderate differentiation, invasion of fiber outer mold. The tumor size was 2.5 × 2 cm and 0.6 × 0.6 cm, and vascular invasion was observed. No cancer involvement was found at the upper and lower incisal margins of the specimen and at the incisal margins of the free anastomosis for examination. A total of 16 lymph nodes were detected without cancer metastasis. Stage IIB (pT3N0M0), immunohistochemical HER2 (‐). In December 2018, the patient received intensity-modulated radiation therapy (PCTV: 50.4 Gy/28F) in the upper mediastinum and bilateral supraclavicular lymphatic drainage area and Tegafur, Gimeracil, and Oteracil Potassium Capsules (referred to as “S1”) (Japan Taiho Pharmaceutical Co., Ltd., Tokushima Plant, State Food and Drug Administration (SFDA) approval number HJ20130811) were taken orally at the same time. In February 2019, the patient returned to the hospital for a follow-up chest CT scan, there was a mass shadow in the upper right lung. Biopsy revealed chronic granulomatous inflammation with scattered caseous necrosis. Therefore, anti tuberculosis treatment was given for 1 year. Therefore, the tuberculosis was clinically cured. In March 2020, due to pain in the right lower limb, the patient returned to the hospital for a follow-up magnetic resonance imaging, which showed multiple abnormal signals in the upper right femur, right pubic branch, and adductor muscle, suggesting metastasis. So the patient was given radiotherapy and PF regimen chemotherapy for 4 courses, supplemented by bone repair, liver protection and other treatments, and the efficacy was PR. On April 2021, the patient experienced pain in the right chest and back, and was given sustained-release morphine sulfate tablets (30 mg q12h) at a local hospital. The pain worsened since 10 days before admission, so sustained-release morphine sulfate tablets were adjusted to 90 mg q12h. Morphine tablets were given to treat the breakthrough pain (Btp). During the use of opioid drugs, the patient experience nausea, dizziness, abdominal distension, abdominal pain, and constipation. In May 2021, he returned to our hospital for further diagnosis and treatment.

### 2.2. Medical history

Having a history of chronic hepatitis B and chronic erosive gastritis for many years, and taking long-term antiviral treatment with entecavir.

### 2.3. Physical examination

Physical condition (Eastern Cooperative Oncology Group) score: 2 scores, numerical rating scale (NRS) 6 scores, clear consciousness, painful expression, old surgical scar visible on the left chest wall, tenderness on the right side of the thoracic vertebrae 8 to 9, no abnormalities on cardiopulmonary auscultation, no tenderness or rebound pain in the abdomen, and no edema in both lower limbs.

### 2.4. Examination data

**Figure 1.**
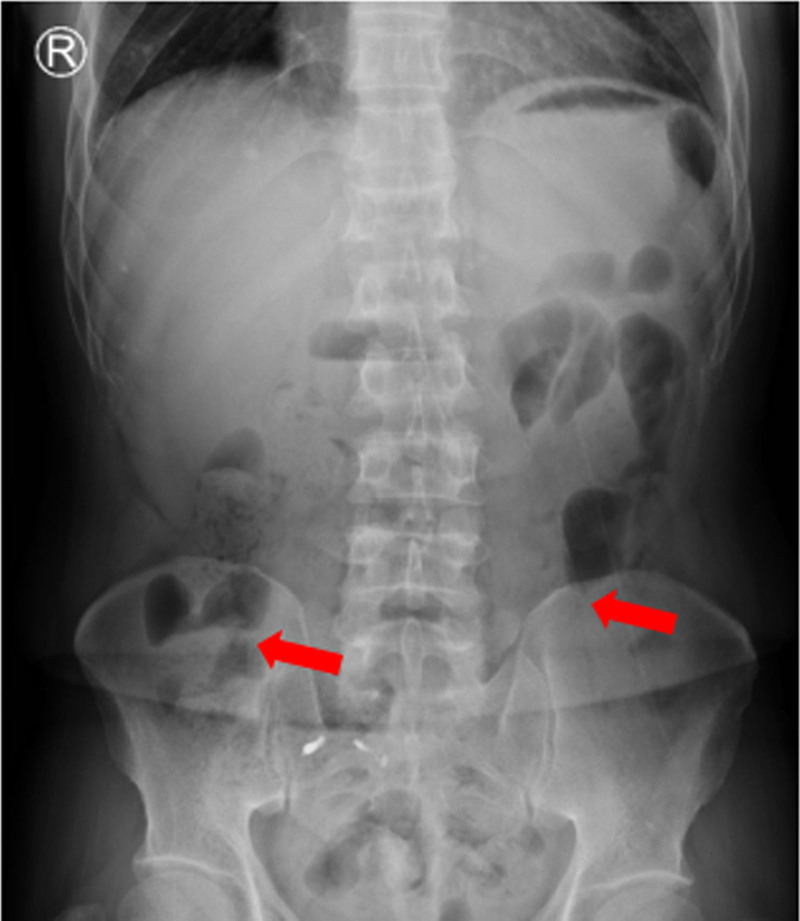
Chest abdominal X-ray showed intestinal gas and fluid accumulation (red arrows).

**Figure 2.**
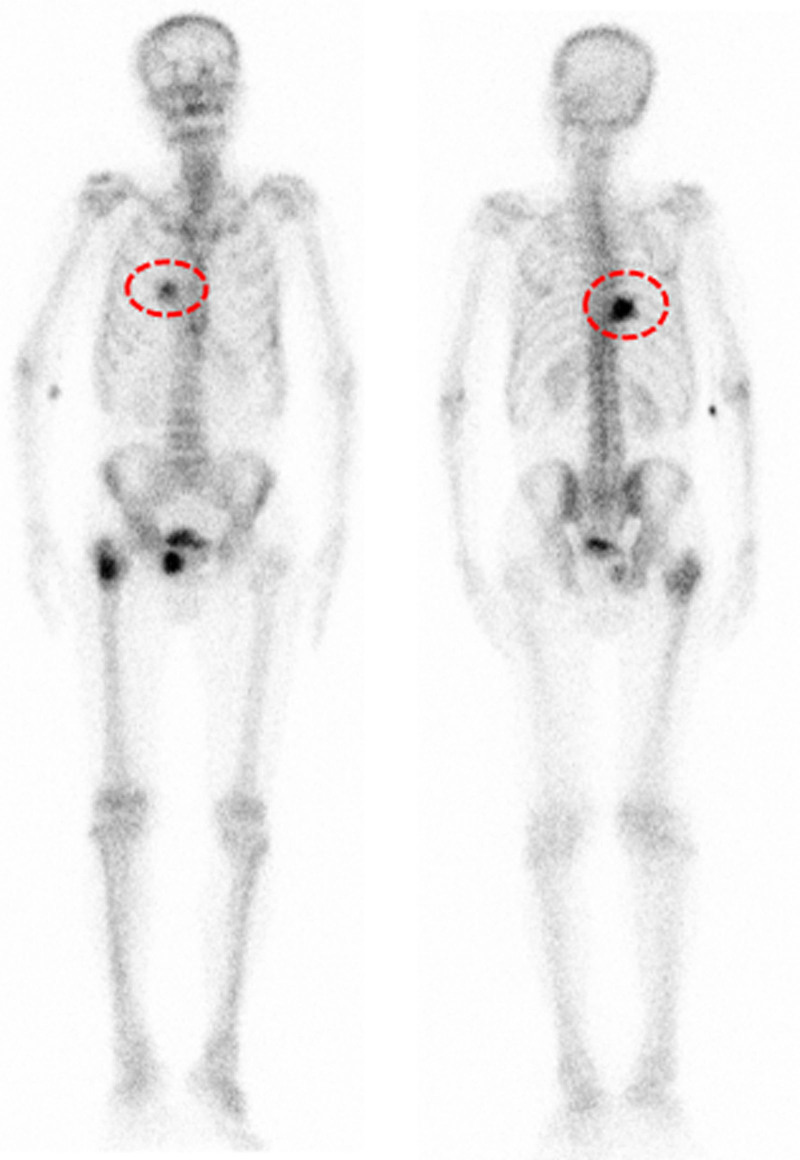
Bone ECT showed multiple bone metastases, with a newly added lesion in the 9th posterior rib on the right side (red circles). ECT = emission computed tomography.

**Figure 3.**
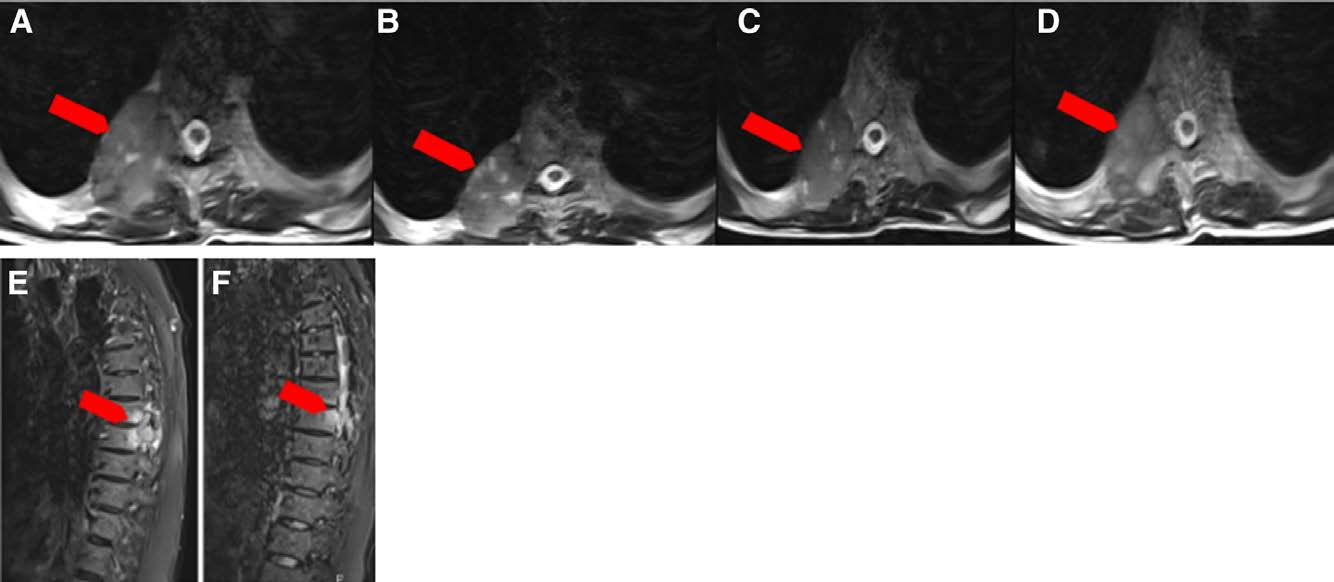
Thoracic MRI showed abnormal bone signals (red arrows) in the right 8th and 9th posterior ribs, T8 and T9 vertebral bodies, and right adnexa, with the formation of a soft tissue (red arrows) mass in the surrounding area side. (A–D) Axial views, (E, F) sagittal views. MRI = magnetic resonance imaging.

(On May 21, 2021) Blood routine showed hemoglobin 91 g/L with no abnormalities in the rest. (On May 21, 2021) Abnormal liver function (ALT 80 U/L, AST 85 U/L) and elevated hepatitis B DNA (3 × 10^3^ copies/mL). (On May 21, 2021) Thoracoabdominal radiography showed intestinal gas and fluid accumulation, and incomplete intestinal obstruction was considered (Fig. 1). (On May 21, 2021) Bone emission computed tomography (Fig. 2) showed increased metabolism in the right ninth posterior rib, upper right femur, and right pubic bone, suggesting tumor bone metastasis. Compared with the previous bone imaging, a new lesion was found in the right ninth posterior rib, and no abnormal bone metabolism was observed in the remaining bones of the body. (On May 22, 2021) The thoracic vertebrae magnetic resonance imaging (Fig. 3) showed abnormal bone signals in the right 8^th^ and 9^th^ posterior ribs, T8 and T9 vertebrae, and right adnexa. Surrounding soft tissue masses were formed, and based on the medical history, metastasis was considered.

### 2.5. Pain assessment

The pain is located on the right chest and back, characterized by bloating, dull pain, intermittent burning like pain, with an NRS score^[12]^ of 6. It is a pain that combines nociceptive pain with neuropathic pain, aggravated during activity or compression, and slightly relieved during rest. Pain treatment history: Administered (30 mg q12h) sustained-release morphine sulfate tablets at a local hospital. The pain worsened since 10 days before admission, so sustained-release morphine sulfate tablets were adjusted to 90 mg q12h, and intermittently administered morphine tablets to treat Btp. Unfortunately, the pain control effect is poor, with poor appetite and sleep, and moderate anxiety.

### 2.6. Clinical diagnosis

(1) Stage IV cardial adenocarcinoma of lower esophagus (rT3N0M1); (2) secondary malignant tumor of bone; (3) cancerous pain (NRS score 6 scores); (4) chronic viral hepatitis B; (5) hepatic insufficiency; (6) incomplete intestinal obstruction; and (7) chronic erosive gastritis.

### 2.7. Analgesic treatment

**Figure 4.**
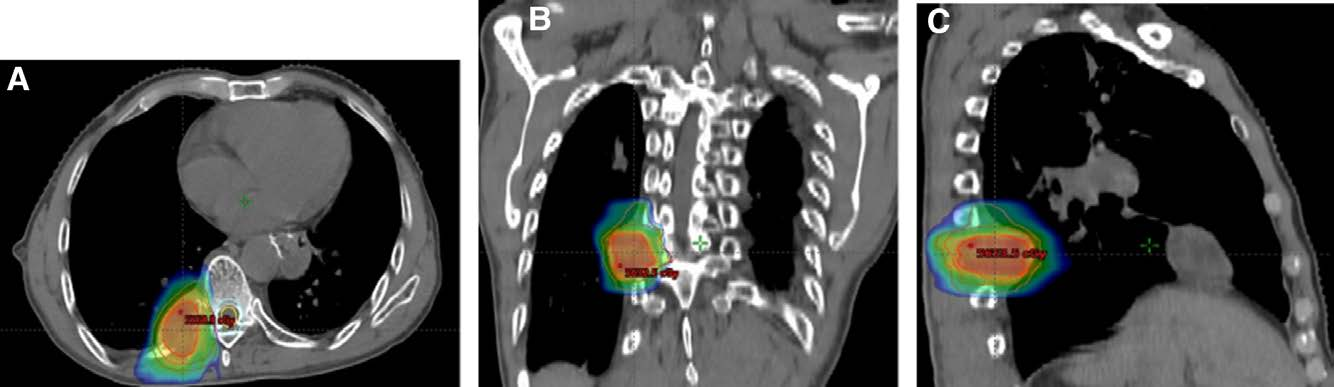
The dose cloud map of the IGRT plan. (A–C) Axial, coronal, and sagittal maps, respectively. IGRT = Image-Guided Intensity Modulated Radiotherapy.

The patient had clinical manifestations of incomplete intestinal obstruction, significant adverse reactions to oral opioid drugs, and poor pain control (NRS 6 scores) during this medical treatment. The patient requestd rapid pain relief. After multidisciplinary team discussion, the patient met the diagnostic criteria for “refractory cancer pain.^[7]^” The main treatment methods for refractory cancer pain^[10]^ include nerve block, epidural administration, PCA analgesia pump, and nerve destruction. Due to the patient’s unwillingness to undergo minimally invasive intervention for pain relief, the WHO revised 4^th^ step PCA pump^[8–11]^ was used for rapid titration, and subcutaneous injection of PCA (referred to as PCSA) was proposed. After stable pain control, the patient switched to fentanyl transdermal patches (Henan Lingrui Pharmaceutical Co., Ltd., SFDA approval number H20163278)^[13–17]^ for maintenance treatment and antitumor etiological treatment. On May 21, 2021 at 11:00, the patient’s oral equivalent dose of morphine for the first 24 hours was 180 mg + 2 × 10 mg = 200 mg. At that time, the NRS score was 6. According to the principle of opioid titration, moderate pain needed to be increased by 25% to 50%, so the amount of morphine required for 24 hours is about 250 to 300 mg. According to the recommendation of the NCCN Adult cancer pain guidelines,^[2]^ when opioid is used in rotation, it should be reduced by 10% to 20% on the basis of the original drug. Therefore, the amount of morphine required for 24 hours was about 200 to 270 mg, oral morphine 60 mg, and hydromorphone injection (produced by Yichang Renfu Pharmaceutical Co., Ltd., SFDA approval number H20120100) 3 mg are essentially equivalent (60 mg oral morphine–3 mg intravenous hydromorphone). Therefore, 24 hours equivalent hydromorphone injection was 10 to 13.5 mg, and PCA pump was prepared according to a preset dose of 1 week, and 100 mg (2 mg/2 mL) of hydromorphone injection and 100 mL of 0.9% sodium chloride injection were added to the pump. So the final concentration of hydromorphone was 0.5 mg/mL. The parameter settings for the subcutaneous pump were as follows: background dose was 13.5 mg/24 h–0.55 mg/h, PCA (Bolus) dose was 13.5 mg/20–0.65 mg/time, and lock time was 15 minutes. Cooperate with treatments such as gastrointestinal decompression, antiemetic (ondansetron), laxative (senna, glycerol enema), nutritional support, anti-infection, psychological counseling,^[18]^ etc. On May 22, 2021 at 11:00 am, NRS score was assessed to be 3 scores, pump was started for 24 hours, bolus was given PCA for 3 times, and the total amount of hydromorphone in 24 hours was as follows: 0.55 mg/h × 24 h + 0.65 mg/time × 3 times = 15.15 mg, a total of 30.3 mL of mixed liquid was pumped, the average NRS score of 24 hours was 3 scores and Btp occurred for 3 times (basically achieve the target of traditional cancer pain treatment). Because Btp occurred for 3 times in the previous 24 hours, the PCA pump’ background dose and bolus dose should have been increased by 20% to 25% on the original basis, but the patients were satisfied. So he required to maintain the current dosage. Meanwhile the patient’s anal exhaust increased, nausea, abdominal distension, dizziness less than before. On May 23, 2021 at 11:00 am, the patient was assessed. After the patient was connected to the pump, the effect began 10 to 15 minutes later, and the NRS score gradually decreased to 2 scores. Btp occurred for 3 times, so bolus was given twice within 24 hours, and the total amount of hydromorphone in the previous 24 hours was as follows: 0.55 mg/h × 24 h + 0.65 mg/time × 2 times = 14.5 mg, a total of 29 mL of mixed liquid was pumped, the average NRS score for 24 hours was 2 scores. The patient was satisfied with no nausea, dizziness, mild abdominal distension, no abdominal pain, and soft stool, so the gastrointestinal decompression tube was removed and combined with anti-distension (dimethicone oil), antineuralgia^[19,20]^ (gabapentin capsule), and antianxiety (estazolam). On May 28, 2021 at 6:00 am, NRS score was assessed as 2 scores in recent days. Bolus was given 1 to 2 times average daily. The pump was about to end, and the patient’s pain control was satisfactory, with an average of 14 mg hydromorphone every 24 hours. The patient was reluctant to continue using PCA pump due to financial difficulties, considering the abnormal liver function of the patient, it was proposed to switch to fentanyl transdermal patch.^[13–17]^ The subcutaneous dosage of hydromorphone every 3 mg is equivalent to 25 µg/h (4.125 mg) fentanyl transdermal patch, so it was necessary to apply fentanyl transdermal patch for about 5.5 patch/72 h, when opioid was used in rotation, the dose should be reduced by 10% to 20%, so we actually applied 5 patch (20.625 mg)/72 h. Table 1 summarizes the entire titration status. The patient continued to receive antiemesis (metoclopramide), defecation (senna or lactulose), antidistention (dimethicone oil), antineuralgia (gabapentin capsule), psychological counseling, and other treatments. Stop using estazolam after the patient’s anxiety was relieved. On May 29, 2021 at 8:00 am, NRS score was 2 scores, Btp occurred once in 24 hours, and was relieved after taking morphine tablets 25 mg orally, and the overall control was satisfactory. There was no nausea, dizziness, abdominal distension, constipation. From May 31 to June 25, 2021, Image-Guided Intensity Modulated Radiotherapy^[21,22]^ (Fig. 4) (PGTV 45Gy/18F, PGTVboost 54Gy/18F) was given to vertebra, adnexa, ribs and soft tissue metastasies, and zolexhospacid 4mg was given to repair bone each time (once every 4 weeks). At the same time, Apatinib mesylate^[23]^ (China Jiangsu Hengrui Pharmaceutical Co., Ltd., National Medical Products approval number H20140103) (500 mg qd) targeted antiangiogenesis therapy was given. After the radiotherapy took effect, fentanyl was gradually reduced. After that, fentanyl transdermal patch (4.125 mg q72h) continued analgesia. The patient was discharged at the end of radiotherapy treatment.

**Table 1:** Medication regimen and titration situation.

Time	Average NRS score before treatment	Method	Average NRS score after treatment	Number of Btp in a 24-h period	Sleep	Anxiety state	Adverse reactions	Management of adverse reactions
First day of titration	6 scores	Hydromorphone 0.55 mg/h, PCA amount 0.65 mg, ondansetron 16 mg/d, psychological counseling	3 scores	3 times	Poor	Moderate	Nausea and constipation	Ondansetron, senna, glycerol enema
Second day of titration	3 scores	Hydromorphone 0.55 mg/h, PCA amout 0.65 mg, ondansetron 16 mg/d, psychological counseling	3 scores	2 times	Slightly poor	Moderate	Mild nausea	Ondansetron, senna
Third day of titration	3 scores	Hydromorphone 0.55 mg/h, PCA amout 0.65 mg, gabapentin 0.3 g qd, estazolam 1 mg qn, psychological counseling	2 scores	2 times	Slightly poor	Moderate	Mild bloating	Dimeticone, senna
Seventh day of titration	2 scores	Hydromorphone 0.55 mg/h, PCA amout 0.65 mg, gabapentin 0.3 g tid, estazolam 1 mg qn, psychological counseling	2 scores	1–2 times	Good	Mild	No adverse reaction	Senna
Opioid rotation maintenance therapy	2 scores	Fentanyl transdermal patch 20.625 mg/72 h, gabapentin 0.3 g tid, estazolam 1 mg qn, psychological counseling	2 scores	1 time	Good	Mild	Mild nausea and bloating	Metoclopramide, dimeticone, senna

Btp = breakthrough pain, NRS = numerical rating scale, PCA = patient-controlled analgesic.

### 2.8. After discharge and follow-up

The patient persisted in using fentanyl transdermal patch (4.125 mg q72h) after discharge. During follow-up, the pain control was satisfactory, the NRS score was 1 to 2 scores. The patient’s mental state, diet, and sleep were normal, and his stool and urine were basically normal, with no intolerable adverse reactions. Regular antitumor systemic treatment was also maintained.

## 3. Discussion

The mechanism of bone metastatic cancer pain includes nociceptive pain, abnormal bone metabolism, changes in the microenvironment of surrounding tissues, activation of nociceptors, nerve compression, and damage caused by tumor growth. Therefore, cancer-related pain associated with bone metastasis usually presents as a mixed type of pain, including nociceptive pain, inflammatory pain, and neuropathic pain, requiring different mechanisms of drug combination analgesia treatment.^[24]^ The patient in this case was diagnosed with advanced lower esophageal and cardial adenocarcinoma with pain in the right side of the chest and back, poorly controlled after the use of opioids in a local hospital, and right side tenderness of thoracic 8 to 9 vertebrae. The pain was characterized by persistent dull pain and intermittent burning pain. Nociceptive pain combined with neuropathic pain caused by direct tumor invasion and compression of local tissue. This was based on a combination of symptoms and imaging data.

According to the history of pain treatment upon admission, the patient used a dose of morphine 180 mg/d (>60 mg/d) for a duration of 10 days (more than 1 week) and was classified as an opioid tolerant^[24]^ patient. After 1 to 2 weeks of standardized drug titration, the patient had poor pain control and severe adverse reactions (nausea, dizziness, abdominal distension, abdominal pain, and constipation), which met the diagnostic criteria of “refractory cancer pain.^[7]^” In addition, the patient had an incomplete intestinal obstruction. The cause of incomplete intestinal obstruction is the side effect of long-term use of opioid drugs and the nonstandard use of laxatives or laxative methods. According to the treatment guidelines^[13]^ and literature reports^[8–11]^ for refractory cancer pain, WHO fourth step interventional minimally invasive therapy or PCA administration should be used. According to the multidisciplinary team discussion opinions and the patient’s wishes, the analgesic treatment program of hydromorphone electronic pump PCSA was adopted, which effectively solved the dilemma that the patient should not be suitable for oral medication at that time. The patient was treated with hydromorphone electronic pump PCSA, combined with gastrointestinal decompression, antineuralgia, anti-emesis, defecation, liver protection, enzyme reduction, antianxiety, psychological nursing, and other treatment measures.

The patient’s pain has gradually been significantly relieved, the frequency of Btp was acceptable. The patient’s sleep and appetite were improved, so that his mental and physical strength have been effectively relieved. In this case, we combined the patient’s pain history (the oral efficacy of morphine was unsatisfactory and the manifestations of incomplete intestinal obstruction such as nausea, abdominal distension, and abdominal pain) with the patient’s urgent need for rapid analgesia, and PCA has the indications of “dosage titration of opioids in cancer pain patients, cancer pain patients with frequent explosive pain, and cancer pain with gastrointestinal dysfunction.^[7]^” This is exactly what the patient needs, so we recommend PCA technology. In addition, based on the results of clinical studies, guideline recommendations^[7]^ and clinical application reports,^[25]^ hydromorphone is suitable for continuous mode administration (intravenous or subcutaneous), and its analgesic titer is superior to morphine. In terms of adverse reactions, the main metabolite of morphine is morphine-3-glucuronic acid (M3G), which has no analgesic effect. Other metabolites morphine-6-glucuronic acid (M6G) and normorphine have analgesic effects, but M6G accumulation in the body will cause adverse reactions. In contrast, hydromorphone metabolites are mainly hydromorphone-3-glucuronic acid (H3G), which has no obvious pharmacological activity. Therefore, the safety of hydromorphone is higher than that of morphine, and the adverse reactions are less than that of morphine, so we recommend hydromorphone. Therefore, the electronic pump PCSA analgesic treatment with hydromorphone is currently a better analgesic option for patients with refractory cancer pain caused by advanced cancer.

In the later stage, the patients fought for the opportunity of antitumor etiological treatment. For pain caused by bone metastases in esophageal cancer, radiation therapy with bone metastases is considered an effective way to relieve pain and to control tumor cells to a certain extent to reduce the risk of pathological fracture.^[21]^ Mohan Hingorani reported that all patients were treated with combined therapeutic strategy using initial systemic chemotherapy followed by local radiotherapy to primary tumor and adjacent areas of visible/residual metastatic disease (metastasis-directed therapy), from the original cohort of metastatic oesophago-gastric cancer patients, 4 cases were identified that developed unusually favorable outcome with long-term survival and probable cure.^[26]^ Based on this, we performed radiation therapy on the patient. As the patient also had local soft tissue metastasis, we prescribed the radiation dose according to the adenocarcinoma radiation dose,^[27]^ rather than solely based on the prescription dose for bone metastasis.^[27,28]^ Finally, he achieved the dual control of symptoms and tumors.

During the pain maintenance treatment phase, we choosed fentanyl transdermal patches with low liver damage and relatively mild gastrointestinal reactions for the patient. Because the metabolite of fentanyl is remifentanil, which has no activity or toxic side effects, there is no need to worry about adverse reactions caused by the accumulation of metabolites during long-term use, the fentanyl is safer. In addition, it should be noted that the reduction principle of opioids is usually reduced gradually according to the principle of 10% to 25%, emphasizing the importance of dynamic assessment. It can be seen from this case that rapid titration of opioids using PCA can quickly relieve refractory cancer pain. This creates an opportunity for subsequent antitumor therapy.

## 4. Conclusion

The clinical treatment of refractory cancer pain presents several challenges, including the complexity of pain mechanisms, individual differences, and factors such as economic and cultural backgrounds, as well as underlying health conditions. These elements significantly influence the choice and effectiveness of cancer pain treatments, especially as personal preferences and economic constraints can restrict clinical decision-making. Individualized comprehensive therapy based on established treatment guidelines or consensus is crucial, with PCA technology playing a pivotal role in the rapid titration of opioids and the management of refractory cancer pain. This approach merits further promotion, particularly in medical institutions in less developed regions.

However, this study is limited by its nature as a single case report, which inherently restricts the sample size and may be influenced by demographic, geographical, or cultural factors that affect the generalizability of the findings. The reliance on researcher observation introduces potential subjective bias. To address these limitations, future research should aim to include multiple cases to enhance sample diversity and result universality. It is essential to clarify the criteria and rationale for case selection, and employ scientific methodologies and tools to ensure data reliability and validity, thereby increasing the research value of the cases.

## Author contributions

**Data curation:** Jiying Tang, Xiaojun Cai.

**Formal analysis:** Jiying Tang.

**Investigation:** Jiying Tang, Mincheng Yu.

**Methodology:** Jiying Tang, Mincheng Yu.

**Project administration:** Xiaojun Cai.

**Supervision:** Xiaojun Cai.

**Writing – original draft:** Jiying Tang, Feng Ding.

**Writing – review & editing:** Feng Ding, Xiaojun Cai.
